# Peiminine Induces G0/G1-Phase Arrest, Apoptosis, and Autophagy *via* the ROS/JNK Signaling Pathway in Human Osteosarcoma Cells *in Vitro* and *in Vivo*

**DOI:** 10.3389/fphar.2021.770846

**Published:** 2021-11-12

**Authors:** Lei Yu, Yuxi Chen, Shaohui Yuan, Yang Cao, Zhenggang Bi

**Affiliations:** ^1^ Department of Orthopedic Surgery, The First Affiliated Hospital of Harbin Medical University, Harbin, China; ^2^ Northern Translational Medicine Research and Cooperation Center, Harbin Medical University, Harbin, China

**Keywords:** peiminine, apoptosis, autophagy, reactive oxygen species ROS, c-Jun N-terminal kinase JNK, osteosarcoma

## Abstract

**Aims:** Peiminine has been reported to have various pharmacological properties, including anticancer activity. In this study, we investigated the effect of this alkaloid on osteosarcoma and explored the underlying mechanisms.

**Methods:** To evaluate the antiosteosarcoma effects of peiminine *in vitro*, cell viability was assessed by CCK-8 and live/dead assays; the effects of the drug on apoptosis and the cell cycle were examined by flow cytometry; the effects on cell migration and invasion were detected by wound healing and Transwell assays, respectively, while its effects on autophagy were observed by transmission electron microscopy and an LC3 fluorescent puncta formation assay. The role of autophagy in the peiminine-mediated effects in osteosarcoma cells was evaluated by CCK-8 assay and western blotting after the application of the autophagy inhibitor chloroquine. The effect of peiminine on reactive oxygen species (ROS) production was analyzed using fluorescence confocal microscopy and spectrophotometry. Additionally, peiminine-treated osteosarcoma cells were exposed to SP600125, a JNK inhibitor, and N-acetylcysteine, a ROS scavenger, after which the contribution of the ROS/JNK signaling pathway to osteosarcoma was assessed using cell viability and LC3 fluorescent puncta formation assays, flow cytometry, and western blotting. A xenograft mouse model of osteosarcoma was generated to determine the antitumor effects of peiminine *in vivo*.

**Results:** Peiminine suppressed proliferation and metastasis and induced cell cycle arrest, apoptosis, and autophagy in osteosarcoma cells. These anticancer effects of peiminine were found to be dependent on intracellular ROS generation and activation of the JNK pathway. In line with these results, peiminine significantly inhibited xenograft tumor growth *in vivo*.

**Conclusions:** Peiminine induced G0/G1-phase arrest, apoptosis, and autophagy in human osteosarcoma cells *via* the ROS/JNK signaling pathway both *in vitro* and *in vivo*. Our study may provide an experimental basis for the evaluation of peiminine as an alternative drug for the treatment of osteosarcoma.

## Introduction

Osteosarcoma (OS), originating from primitive osteogenic mesenchymal cells, is the most frequently diagnosed primary malignant bone tumor. Approximately 2–3 people per million, mainly adolescents and young adults, are diagnosed with OS each year (Wang et al., 2016a; Zhu et al., 2017). Although novel adjuvant chemotherapy regimens and improved surgical techniques have extended the 5-years survival rate of patients to approximately 70% (Liu et al., 2017), the cure rate has remained stagnant over the last 30 years (Wang et al., 2019). This underlines the need to identify new, safer, and more efficient drugs to treat this disease (Chen et al., 2019).

Cell cycle progression is regulated *via* the activity of cell cycle protein-dependent kinases (CDKs) and CDK inhibitory proteins (Wang et al., 2016b). Dysregulation of the cell cycle is associated with tumor initiation and progression and is a hallmark of cancer cells (Stewart et al., 2003). Many anticancer drugs can induce G0/G1-phase arrest by targeting cell cycle-related pathways (Li et al., 2015a; Wang et al., 2016a).

Apoptosis, or type I programmed cell death, is the classical pathway underlying chemotherapeutic drug-induced cancer cell death (Nishida et al., 2008; Ouyang et al., 2012; Ravegnini et al., 2017). Autophagy, represents a crucial mechanism for the maintenance of cellular homeostasis *via* the recycling of cellular components. Studies have demonstrated that autophagy has a dual role in tumor development, both suppressing early tumorigenesis and promoting survival and growth in advanced cancers (Tanaka et al., 2016; Cerezo and Rocchi, 2017). However, autophagy was found to be associated with apoptosis (Fu et al., 2020).

It has been reported that the cellular redox state is related to both oncogenesis and antitumor processes (Sabharwal and Schumacker, 2014; Raj et al., 2015). Reactive oxygen species (ROS) are byproducts of aerobic metabolism (Scherz-Shouval and Elazar, 2011). Moderately elevated ROS levels can contribute to cell proliferation and differentiation, whereas ROS overload can result in oxidative damage in tumor cells through the triggering of apoptotic and autophagic processes (Anozie and Dalhaimer, 2017; Kalai Selvi et al., 2017). ROS have also been shown to directly or indirectly activate members of the MAPK family, including p38, c-Jun N-terminal kinase (JNK), and extracellular regulated protein kinases1/2 (ERK1/2) (Shen and Liu, 2006). JNK is a crucial modulator of multiple cellular events, and focusing on related signaling pathways, especially the ROS/JNK signaling pathway, has potential as an alternative therapeutic strategy for the treatment of OS.

Peiminine is an alkaloid derived from *Fritillaria thunbergii* and is widely used in Chinese medicine for the treatment of numerous diseases, including cancer (Lyu et al., 2015); however, whether peiminine also has ameliorative effects in human OS has not been explored. In this study, we evaluated the antiosteosarcoma effects of peiminine *in vitro* and *in vivo*. Specifically, we investigated whether peiminine treatment could promote apoptosis or autophagy in OS cells, as well as the relationship between peiminine-induced apoptosis and autophagy. Our results showed that peiminine induced G0/G1-phase arrest, apoptosis, and autophagy through the ROS/JNK pathway in OS, suggesting that peiminine has potential as a therapeutic agent for the treatment of OS.

## Materials and Methods

### Cell Lines and Cell Culture

The human osteosarcoma cell lines MG-63 and Saos-2 were cultured in Dulbecco’s modified Eagle’s medium (DMEM; Gibco, United States) and McCoy’s 5A medium (Procell, China) respectively supplemented with 10% fetal bovine serum (FBS) (Gibco). All the cells were cultured in an incubator at 37°C with 5% CO_2_.

### Antibodies and Reagents

Antibodies targeting the following proteins were used in this study: cyclin D1 (WL01435a), CDK2 (WL01543), p27 (WL04174), cleaved caspase-9 (WL01838), cleaved caspase-3 (WL01992), Bcl-2 (WL01556), Bax (WL01637), LC3BII/I (WL01506), beclin-1 (WL02508), p62 (WL02385), p-JNK (WL01813), JNK (WL01295), c-JUN (WL02863), E-cadherin (WL01482), vimentin (WL01960), MMP-2 (WL1579), MMP-9 (WL01580), β-actin (WL01372), goat anti-rabbit IgG-HRP (WLA023) (all from Wanleibio, China), and p-c-JUN (AF3095, Affinity, United States). Purified peiminine (98%) was obtained from Yuanye Bio Co., Ltd (Shanghai, China; B20081). Cell Counting Kit-8 (CCK-8; C0037), the live/dead assay kit, the cell cycle and apoptosis analysis kit, the ROS Kit, NAC, SP600125, and the terminal dUTP nick-end labeling (TUNEL) Kit (C1086) were acquired from Beyotime Institute of Biotechnology (Shanghai, China). Tetramethylrhodamine methyl ester (TMRM; I34361) and Hoechst 3342 (H3570) were sourced from Life Technology (OR, United States). The mRFP-GFP-LC3 adenovirus was acquired from HanBio Technology Co., Ltd (Shanghai, China). The rest of the reagents and experimental materials were purchased from common commercial sources.

### Cell Viability Assay

MG-63 and Saos-2 cells were treated with different concentrations of peiminine for 24, 48, or 72 h. CCK-8 solution was then added to the cells following the manufacturer’s protocol and incubated for an additional 2 h at 37°C. The absorbance at 450 nm was calculated using a microplate reader (ELX-800, BioTek, China).

### Live/Dead Assay

Cells were incubated in a medium containing 2.5 μM calcein AM and 4 μM ethidium homodimer 1 (EthD-1) (C2015M, Beyotime) according to the manufacturer’s instructions. Cells were observed and counted under a fluorescence microscope (U-RFL-T, Olympus, Tokyo, Japan).

### Flow Cytometric Analysis of the Cell Cycle and Apoptosis

For cell cycle analysis, cells were collected and fixed in 75% ethanol at 4°C overnight. The next day, the cells were incubated in staining buffer for 15 min and then resuspended. Propidium iodide (PI) and ribonuclease (RNase) (C1052, Beyotime) were subsequently added and the cells were cultured in the dark for 30 min. Finally, the proportion of cells in each phase of the cell cycle was determined by flow cytometry (Becton Dickinson, United States).

For the analysis of apoptosis, Annexin V-FITC/PI double-staining kit (C1062L, Beyotime) was employed. Peiminine-treated cells were harvested, resuspended in 1 × binding buffer, and incubated with Annexin V-FITC and PI in the dark for 15 min according to the manufacturer’s protocol. Finally, the cells were examined by flow cytometry (Becton Dickinson).

### Measurement of Mitochondrial Membrane Potential

Cells were cultured in glass-bottom cell culture dishes and pretreated with different concentrations of peiminine. After discarding the original culture medium, the cells were rinsed with phosphate-buffered saline (PBS) and incubated with TMRM at 37°C with 5% CO_2_ for 45 min. After discarding the medium, the cells were rinsed with PBS, incubated with Hoechst 3342 for 15 min, and imaged using a fluorescence confocal microscope (FV10CW3, Olympus). The fluorescence intensity representing the mitochondrial membrane potential was measured using ImageJ software (National Institutes of Health, United States).

### Transmission Electron Microscopy

Transmission electron microscopy (TEM) was employed to observe the effect of peiminine on cell ultrastructure. Cells were fixed in 2.5% glutaraldehyde and post-fixed in 1% osmium tetroxide. After dehydration with different concentrations of ethanol, the cells were embedded in Epon. Ultrathin sections were observed under a transmission electron microscope (Hitachi TEM system).

### Evaluation of Fluorescent LC3 Puncta

OS cells were transfected with mRFP-GFP-LC3 adenovirus. Fluorescent puncta formed by autophagosomes (yellow) and autolysosomes (red) represented cell autophagy. After 24 h of transfection, the cells were treated with peiminine for 48 h, and then viewed and imaged using a fluorescence confocal microscope (FV10CW3, Olympus).

### Reactive Oxygen Species Assay

Following the manufacturer’s instructions, cells were first incubated with the DCFH-DA probe (S0033M, Beyotime) for 20 min at 37°C, following which the nuclei were counterstained with Hoechst 3342 for 15 min. Finally, the cells were viewed using a fluorescence confocal microscope (FV10CW3, Olympus). Fluorescence absorbance was measured using an Infinite 200 PRO microplate reader (Tecan, Switzerland).

### Western Blotting Analysis

Cells were cultured as described above and treated. The cells were lysed on ice with cold radioimmunoprecipitation (RIPA) lysis buffer containing 1% protease inhibitor and 10% phosphatase inhibitor for 30 min. After centrifugation at 4°C for 15 min at 13,500 rpm, the supernatants containing total protein contents were collected and the protein levels were quantified using the BCA Protein Assay Kit (P0009, Beyotime). Equal amounts of total protein were separated by SDS–PAGE (8–12%) and then transferred onto nitrocellulose membranes. After blocking with 5% skimmed milk for 2 h, the membranes were first incubated (with shaking) with primary antibodies at 4°C overnight and then with the respective fluorescently labeled secondary antibodies at room temperature in the dark for 1 h. Finally, the target bands were detected using the Odyssey Infrared Imaging System (LI-COR Biosciences, United States).

### Wound Healing Assay

Confluent cells were treated with peiminine for 48 h. A wound (scratch) was made in the cells using a 100 μL pipette tip, and the detached cells were washed off with serum-free medium and then cultured with 10% FBS (Jin et al., 2020). Wound healing was assessed at different time points (0, 12, and 24 h) under a microscope (CKX41, Olympus).

### Transwell Assay

Matrigel (356234, BD, United States) was precoated on the upper layer of the Transwell chamber (3422, Corning, United States), after which 200 μL of a serum-free cell suspension (2 × 10^4^ cells that had been pretreated with different concentrations of peiminine for 48 h) was added to the upper chamber and 700 μL of medium containing 30% FBS was placed in the lower chamber. After 12 h, cells in the lower chamber (migrated cells) were fixed in 4% paraformaldehyde at room temperature for 20 min and stained with crystal violet for 15 min. Finally, images were acquired under an inverted microscope (TH4-200, Olympus) at × 200 magnification. The numbers of migrated cells were counted using ImageJ software.

### Xenograft Osteosarcoma Mouse Model

Female BALB/c nude mice (4 weeks old) were acquired from the Shanghai Experimental Animal Center of the Chinese Academy of Sciences. MG-63 cells (1 × 10^6^ cells in, 100 μL of PBS) were subcutaneously implanted into each mouse. One week after implantation, all the mice were randomly divided into two groups (*n* = 6 per group). Mice in the control group were administered 100 μL of vehicle by intraperitoneal injection every other day, while mice in the peiminine treatment group were administered 100 μL of peiminine (2 mg/ kg) (Zhao et al., 2018) by intraperitoneal injection every other day. After seven doses (21 days after tumor implantation), all the mice were euthanized and the tumors were separated, weighed, and fixed for subsequent analysis.

### TUNEL Assay

In brief, sections were deparaffinized, hydrated, incubated with proteinase K, permeabilized, incubated with freshly prepared TUNEL reaction mixture, and then with DAPI (C1006, Beyotime) for 5 min in the dark. The sections were imaged under a microscope (U-HGLGPS, Olympus) at × 200 magnification.

### Histopathology and Immunohistochemistry

Tissue specimens were fixed, embedded, and serially sectioned (4 μm). The primary tumor and major organs (heart, liver, spleen, lungs, and kidneys) were stained with hematoxylin and eosin (H&E) for morphological evaluation. For immunohistochemical staining, sections were dewaxed with xylene and dehydrated using a graded ethanol series. After antigen retrieval in 10 mM sodium citrate, the sections were blocked in 5% BSA, incubated with primary antibodies overnight at 4°C, and then with the appropriate secondary antibodies at room temperature for 1 h. Immunoreactivity was detected using a DAB Kit (P0203, Beyotime).

### Statistical Analysis

GraphPad Prism version 8.0 (GraphPad Software Inc., La Jolla, CA, United States) was used for statistical analysis. All data are shown as means ± SD of independent experiments, each repeated at least three times. Tukey’s multiple comparison test or one-way ANOVA was applied to determine whether there were any statistically significant differences among the different groups. A *p*-value < 0.05 was considered statistically significant.

## Results

### Peiminine Inhibited the Proliferation of OS Cells

**FIGURE 1:**
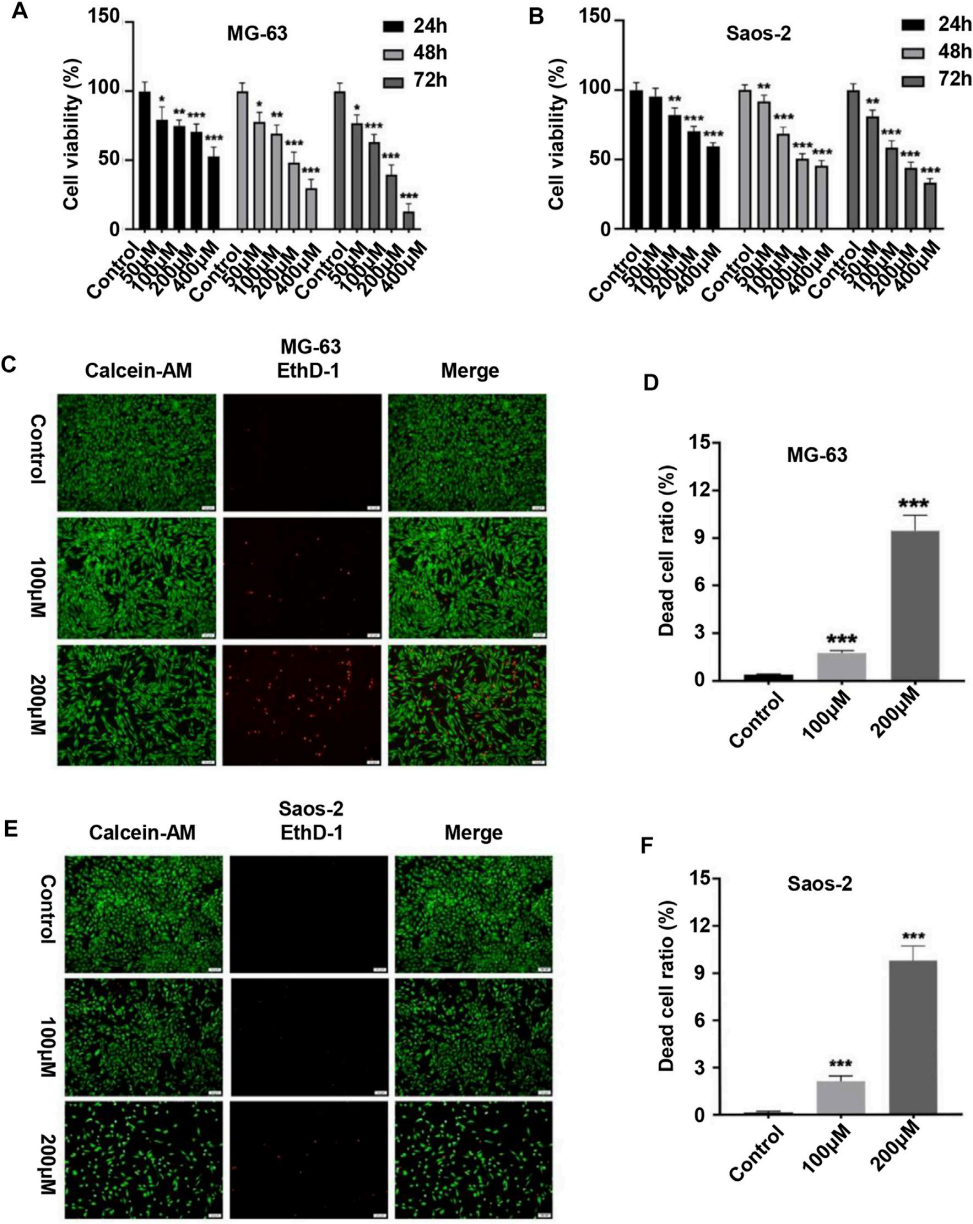
Peiminine inhibited the proliferation of osteosarcoma cells. **(A,B)** Human osteosarcoma MG-63 and Saos-2 cells were treated with peiminine (0–400 μM) for 24, 48, or 72 h, following which cell viability was assessed by cell counting kit-8 (CCK-8) assay. **(C,E)** Peiminine significantly reduced the number of live cells (green fluorescence) and increased the number of dead cells (red fluorescence) in a concentration-dependent manner after 48 h of treatment as determined under an inverted fluorescence microscope. Scale bar = 100 μm. **(D,F)** Quantitative analysis of the live and dead assays (*n* = 3). *p < 0.05, **p < 0.01, ***p < 0.001.

We first evaluated the effect of peiminine on the proliferative ability of OS cells. MG-63 and Saos-2 cells were treated with various concentrations of peiminine for 24, 48, and 72 h, following which cell viability was measured by CCK-8 assay. The results showed that peiminine significantly inhibited the proliferation of OS cells *in vitro* in a time- and dose-dependent manner (Figures 1A,B). Treatment with 100 and 200 μM peiminine for 48 h was selected for subsequent experiments based on the IC_50_ values obtained from the CCK-8 assays (Supplementary Table S1). The live/dead assay also showed that peiminine treatment disrupted the morphology of OS cells and led to a significant increase in the number of dead cells compared with the control group (Figures 1C–F). These results indicated that peiminine exerts a significant inhibitory effect on the proliferative ability of OS cells in a time- and dose-dependent manner.

### Peiminine Induced Cell Cycle Arrest in OS Cells at the G0/G1 Phase

**FIGURE 2:**
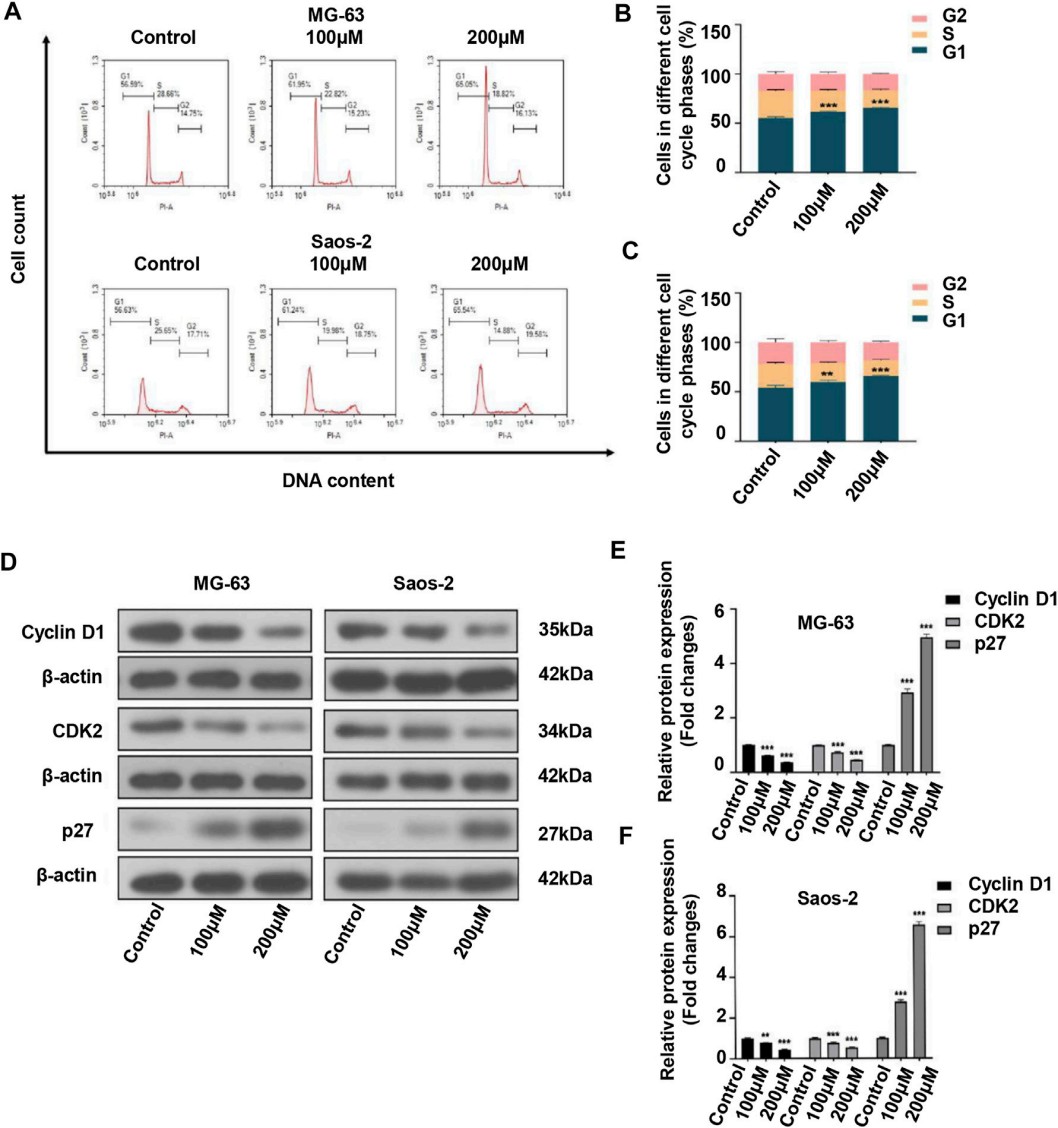
Peiminine induced cell cycle arrest at the G0/G1 phase in osteosarcoma cells. **(A**–**C)** Peiminine treatment promoted G0/G1 cell cycle arrest as determined by flow cytometry. **(D)** Western blot analysis of the expression of the cell cycle-associated proteins cyclin D1, CDK2, and p27. **(E,F)** Quantitative analysis of the levels of cell cycle-associated proteins (*n* = 3). β-actin served as the control. **p < 0.01, ***p < 0.001.

Considering that cell cycle arrest can suppress cell proliferation, we investigated the effect of peiminine on the cell cycle of OS cells. The results showed that the proportion of OS cells in the G1 phase was significantly increased after peiminine treatment compared with that in the control group (Figures 2A–C). In addition, the expression of key regulatory factors involved in the G0/G1-phase transition was significantly changed. The levels of cyclin D1 and CDK2 were increased, whereas those of p27 were decreased (Figures 2D–F). In summary, these findings indicated that peiminine induces cell cycle arrest at the G0/G1 phase through regulating the levels of cell cycle-related proteins in OS cells.

### Peiminine Induced Apoptosis in OS Cells

**FIGURE 3:**
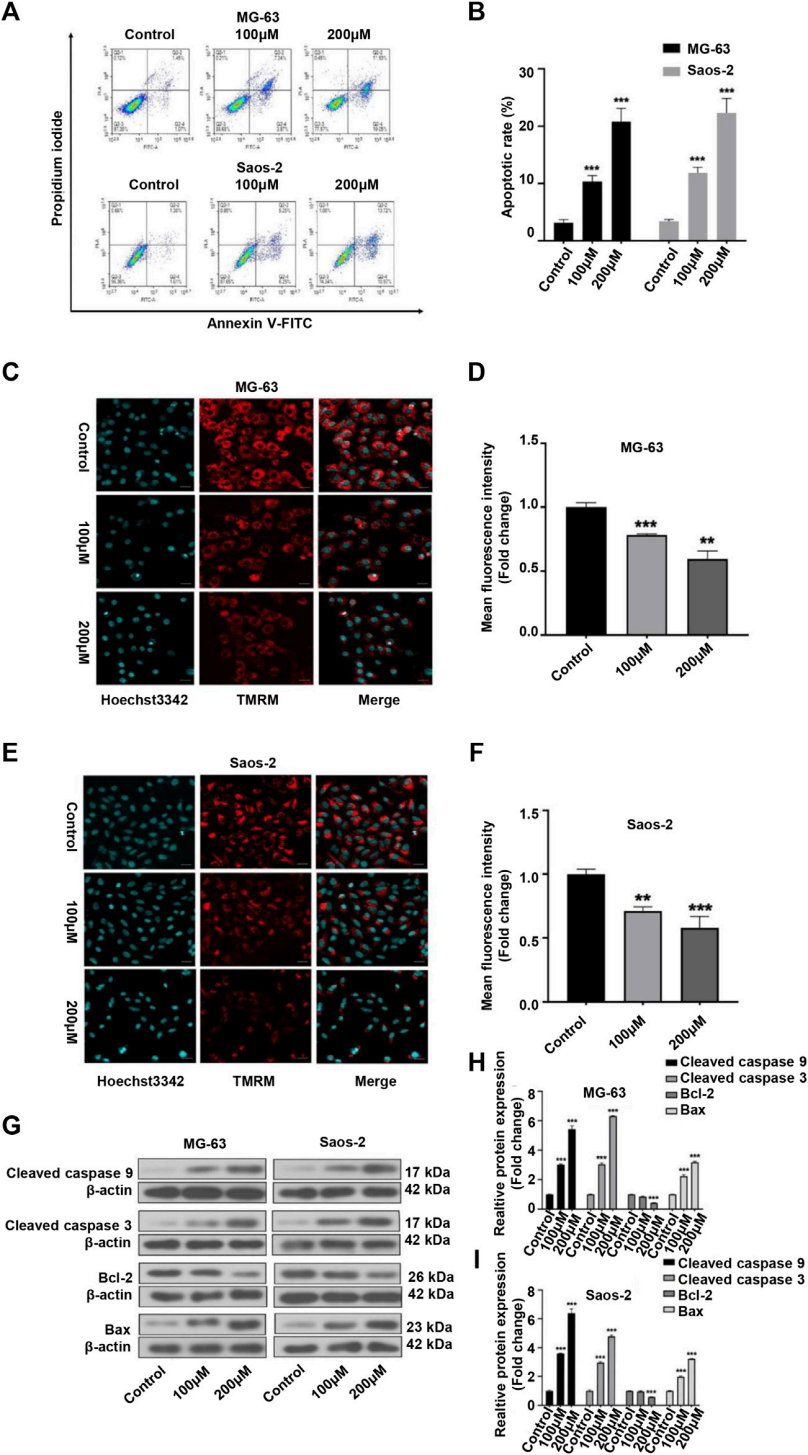
Peiminine induced apoptosis in osteosarcoma (OS) cells. **(A,B)** OS cells were stained with Annexin V-FITC/PI and the percentage of apoptotic cells was determined by flow cytometry. **(C,E)** Images of tetramethylrhodamine methyl ester (TMRM) staining showing that peiminine treatment led to the depolarization of the mitochondrial membrane. Scale bar = 20 μm. **(D,F)** Quantitative analysis of the mitochondrial membrane potential in OS cells. **(G)** Western blot was used to examine the expression of cleaved caspase-9, cleaved caspase-3, Bcl-2, and Bax in OS cells. β-actin was used as control. **(H,I)** Quantitative analysis of the levels of apoptosis-related proteins (*n* = 3). **p < 0.01, ***p < 0.001.

As many anticancer drugs exert their effects through activating the apoptotic pathway (Baig et al., 2016), we next examined whether the inhibitory effects of peiminine on OS were associated with apoptosis. Annexin V-FITC/PI staining showed that the apoptosis rate of OS cells was significantly higher with than without peiminine treatment (Figures 3A,B). Because the loss of mitochondrial membrane potential is known to be a critical step in apoptosis (Fu et al., 2020), we evaluated the impact of peiminine treatment on the mitochondrial membrane potential in OS cells as well as the expression of essential proteins involved in the mitochondrial apoptosis pathway. The results showed that the mitochondrial membranes of OS cells underwent significant depolarization after peiminine treatment (Figures 3C–F). Furthermore, the levels of the mitochondrial apoptosis pathway-related proteins caspase-9, caspase-3, and Bax were significantly upregulated, whereas those of Bcl-2 were decreased (Figures 3G–I). These observations suggested that peiminine induces OS cell apoptosis *via* the mitochondrial pathway.

### Peiminine Induced Autophagy in Osteosarcoma Cells

**FIGURE 4:**
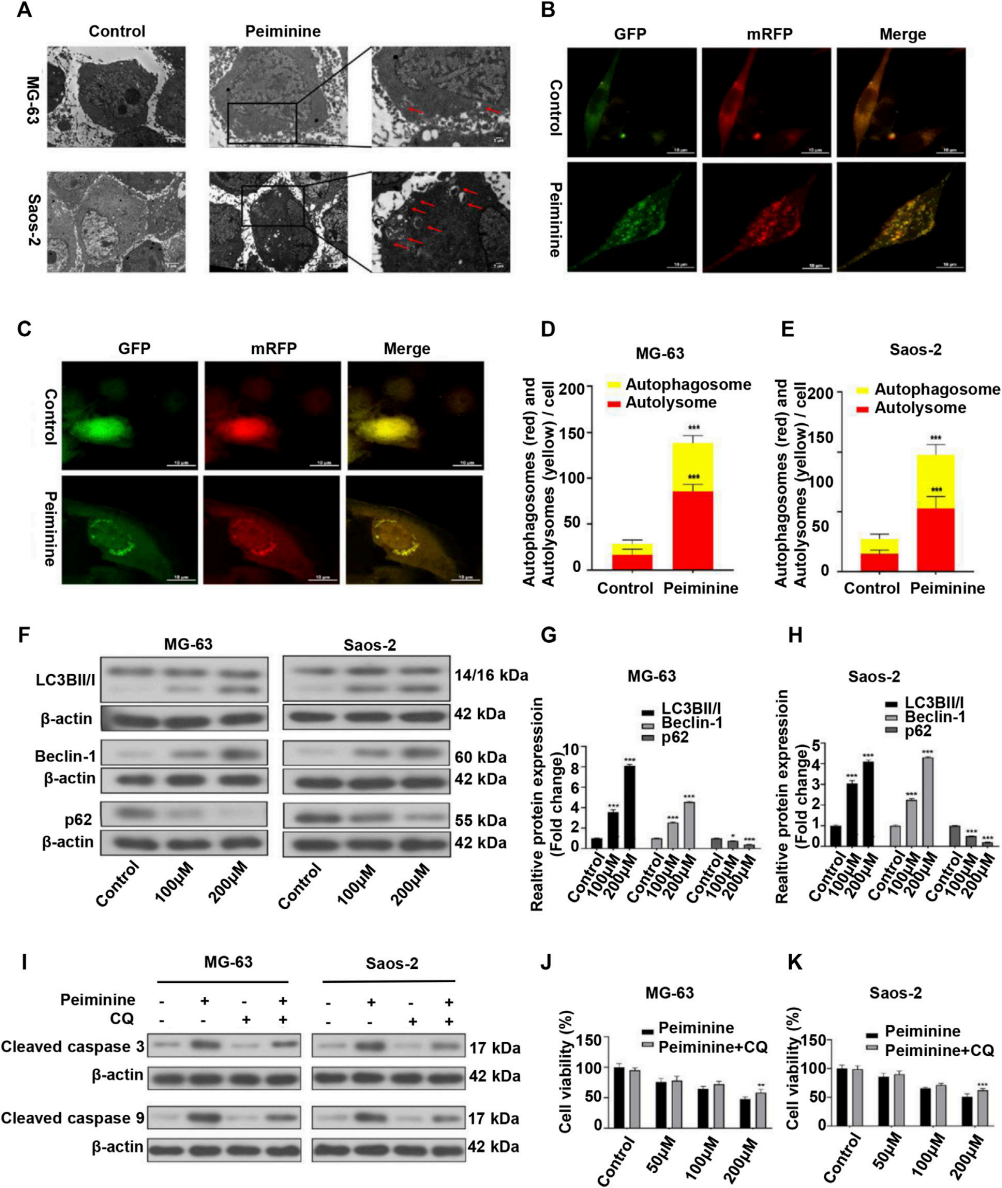
Peiminine induced autophagy in osteosarcoma (OS) cells. **(A)** Transmission electron micrographs showing autophagosome formation. Autophagosomes are indicated by arrows. Control, Lower scale bar = 5 μm, Upper scale bar = 2 μm. **(B,C)** Peiminine induced autophagic flux. Scale bar = 10 μm. **(D,E)** Quantitative analysis of autophagic flux in OS cells. **(F**–**H)** OS cells were treated with peiminine at various concentrations for 48 h. The expression levels of the autophagy-related proteins LC3B II/I, beclin-1, and p62 were measured by western blot (*n* = 3). β-actin served as control. **(J,K)** OS cells were pretreated with chloroquine (CQ, 20 μM) for 2 h and then treated with peiminine for 48 h. Cell viability was assessed by cell counting kit-8 (CCK-8) assay. **(I)** The levels of apoptosis-related proteins were measured by western blot after peiminine treatment in the presence or absence of CQ. *p < 0.05, **p < 0.01, ***p < 0.001.

Autophagy has been implicated in tumor cell death (Li et al., 2020). Here, we investigated whether the inhibitory effect of peiminine on OS cells was partly mediated *via* autophagic processes. Compared with the control group, the numbers of autophagosomes were significantly increased after peiminine treatment as determined by TEM (Figure 4A). Consistent with these results, we observed that the numbers of autophagosomes and autolysosomes were both significantly increased in OS cells transfected with mRFP-GFP-LC3 adenovirus (Figures 4B–E). Additionally, western blotting analysis showed that peiminine treatment led to an increase in the expression of LC3B II and beclin-1 and a decrease in that of p62 in OS cells (Figures 4F–H). Taken together, these results indicated that peiminine induced the formation of autophagosomes and promoted autophagic flux in OS cells. In the context of cancer treatment, it is known that autophagy can promote either tumor cell survival or tumor cell death. To determine which effect applied to peiminine treatment, we exposed OS cells to chloroquine (CQ), an autophagy inhibitor (Duffy et al., 2015). We found that CQ (20 μM) could attenuate the inhibitory effect of peiminine on OS proliferation (Figures 4J,K). Meanwhile, western blotting results showed that CQ suppressed the peiminine treatment-mediated upregulation of the levels of apoptosis-related proteins (Figure 4I). These findings suggested that peiminine-induced autophagy promotes apoptosis and cell death in OS.

### Peiminine Induced ROS Production and Activated the JNK Signaling Pathway

**FIGURE 5:**
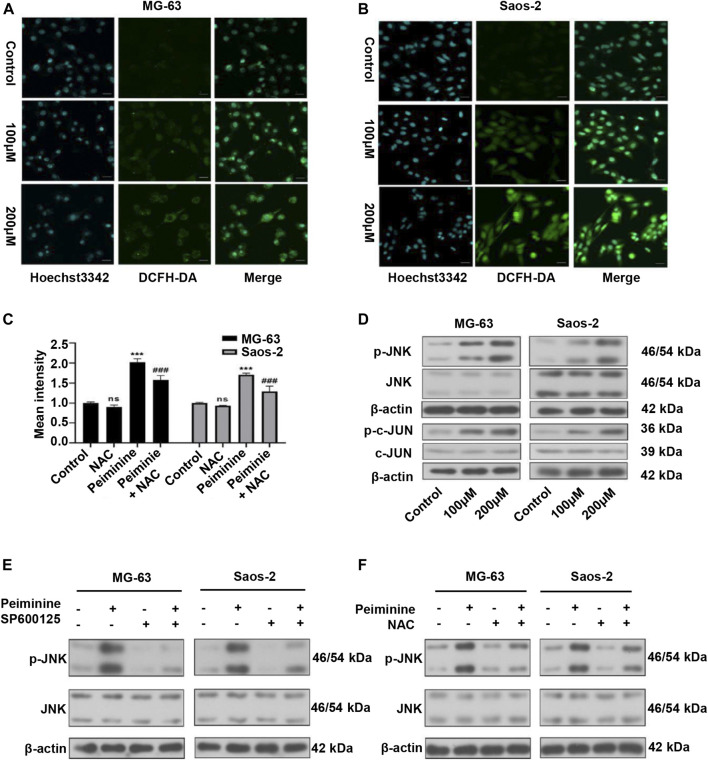
Peiminine induced reactive oxygen species (ROS) generation and activated the ROS/JNK signaling pathway in osteosarcoma (OS) cells. **(A,B)** Representative images of intracellular ROS levels as detected by fluorescence microscopy following DCFH-DA staining. Scale bars = 20 μm. **(C)** Intracellular ROS levels were measured after treatment with peiminine (200 μM) in the presence or absence of NAC (*n* = 3). ***p < 0.01 *vs*. control, ^###^ *p* < 0.001 *vs*. peiminine treatment. **(D)** The expression of p-JNK, JNK, p-c-Jun, and c-Jun was analyzed by western blot after treatment with peiminine. **(E,F)** OS cells were preincubated with SP600125 (30 μM) or NAC (5 mM) for 2 h and then treated with peiminine for 48 h. Related protein expression was analyzed by western blotting.

We have previously shown that ROS have a major impact on tumor cell fate (Zou et al., 2017). In this study, we assessed ROS intracellular levels using a DCFH-DA probe (Figures 5A,B; Supplementary Figure S1). The results showed that peiminine (200 μM) promoted a significant increase in intracellular ROS levels in OS cells, whereas NAC application could block this effect (Figure 5C). The JNK signaling pathway is a known ROS target (Wang et al., 2016a). Here, we found that peiminine could activate the JNK pathway in OS cells (Figure 5D). Moreover, both SP600125 (30 μM) and NAC (5 mM) treatment could prevent the peiminine-induced activation of the JNK pathway (Figures 5E,F). Taken together, these results demonstrated that peiminine activates the ROS/JNK axis in OS cells.

### Peiminine Induced Apoptosis and Autophagy Through the Activation of the ROS/JNK Signaling Pathway

**FIGURE 6:**
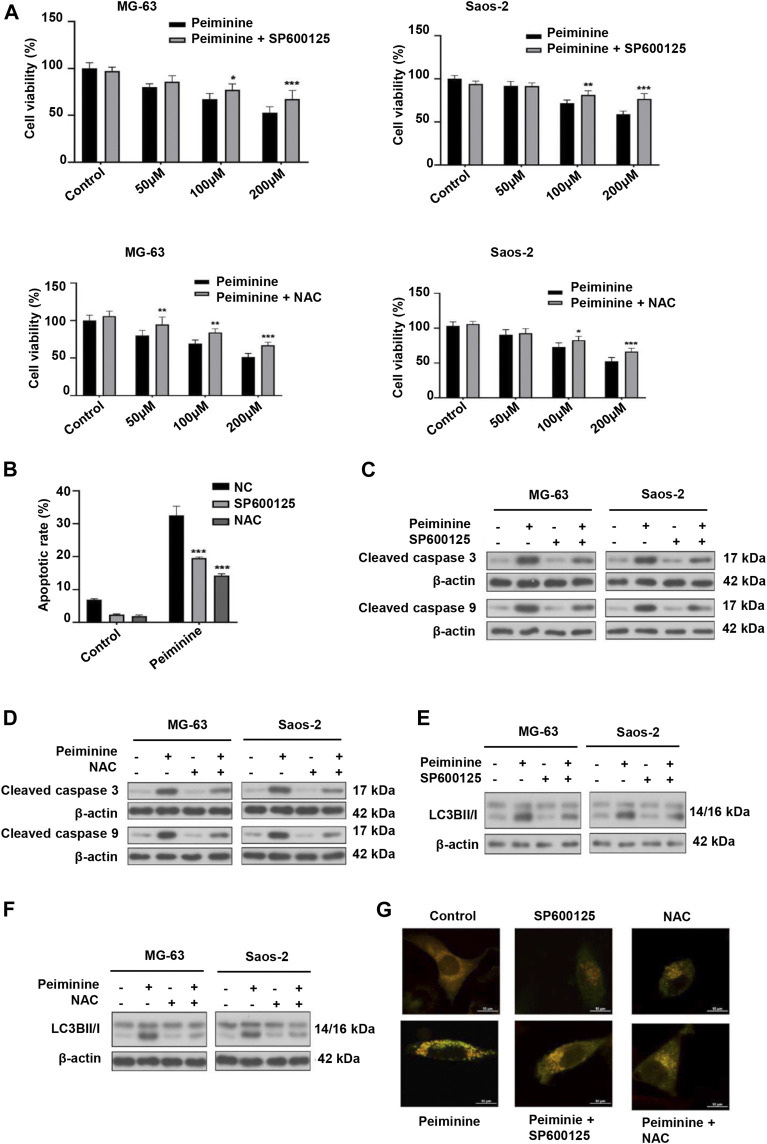
Peiminine induced cell apoptosis and autophagy *via* the activation of the ROS/JNK signaling pathway. Osteosarcoma (OS) cells were pretreated with SP600125 (30 μM) or NAC (5 mM) for 2 h and then treated with peiminine (200 μM) for 48 h. **(A)** Cell viability was measured by cell counting kit-8 (CCK-8) assay (*n* = 3). **(B)** Apoptotic cells were detected by flow cytometry (*n* = 3). **(C,D)** The expression levels of apoptosis-related proteins and **(E,F)** autophagy-related proteins were measured by western blot. **(G)** Representative images of OS cells showing the mRFG-GFP-LC3 puncta. OS cells were pretreated or not with SP600125 or NAC and then incubated with peiminine or vehicle. Scale bar = 20 μm. *p < 0.05, **p < 0.01, ***p < 0.001.

Next, we investigated whether peiminine-induced apoptosis and autophagy in OS cells were dependent on the ROS/JNK pathway. We found that exposure to either SP600125 or NAC could attenuate the inhibition of OS cell proliferation mediated by peiminine as determined by the CCK-8 assay (Figure 6A). Flow cytometric analysis further indicated that SP600125 (30 μM) or NAC (5 mM) treatment prevented the peiminine-induced (200 μM) increase in the apoptotic rate in OS cells (Figure 6B), while western blot results demonstrated that both treatments also suppressed the expression of proapoptotic-related proteins (Figures 6C,D). Additionally, in peiminine-treated OS cells, the number of mRFP-GFP-LC3 puncta and the levels of autophagy-associated proteins were also reduced following SP600125 and NAC treatment (Figures 6E–G). Taken together, these results suggested that peiminine induces OS cell apoptosis and autophagy through the ROS/JNK signaling pathway.

### Peiminine Suppressed the Migration and Invasion of Osteosarcoma Cells

**FIGURE 7:**
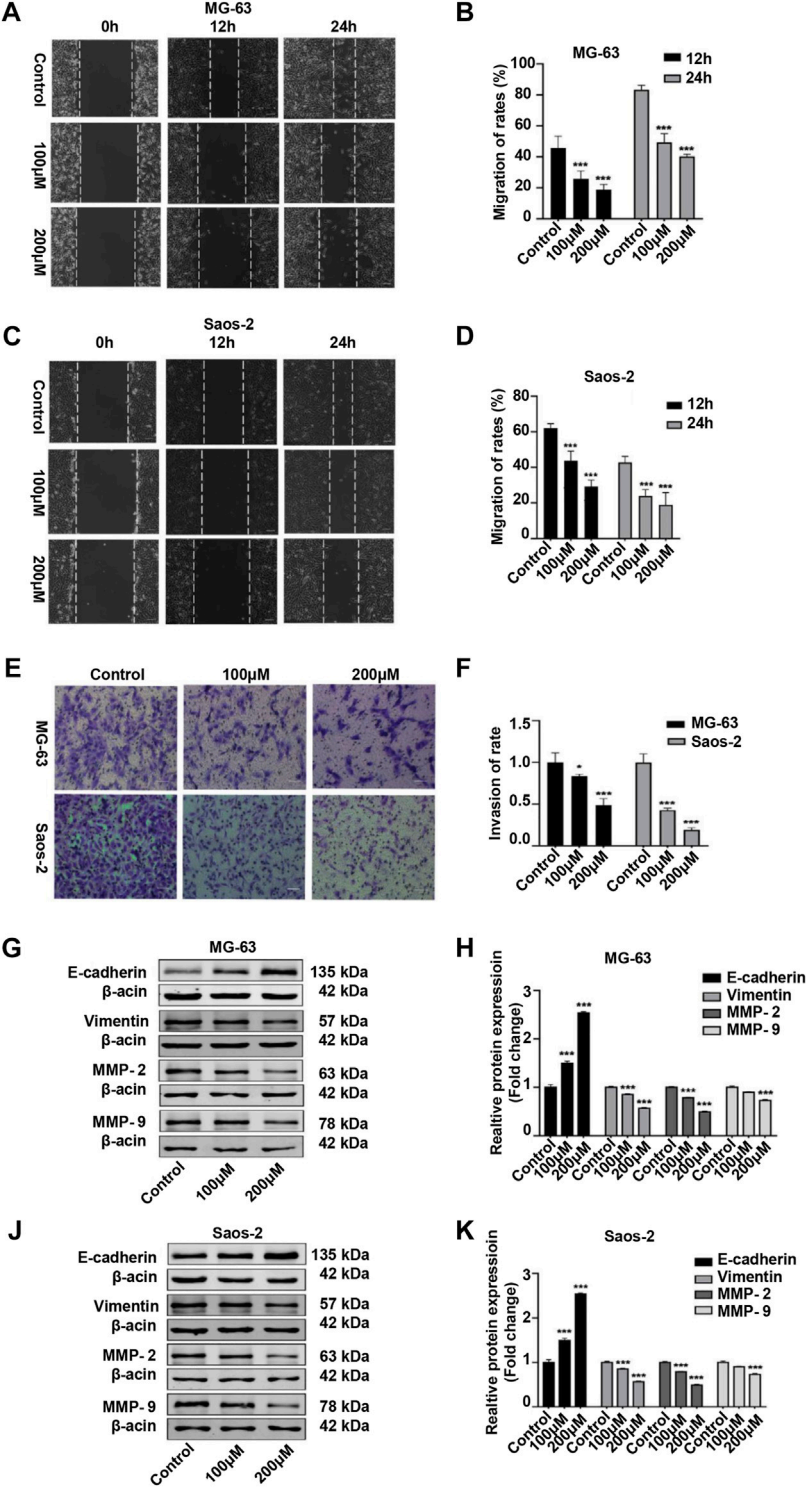
Peiminine suppressed migration and invasion of osteosarcoma (OS) cells. **(A,C)** Wound healing assay in OS cells treated with peiminine for 0, 12, or 24 h. Scale bar = 200 μm. **(B,D)** Quantitative analysis of the OS cell migration rate (*n* = 3). **(E)** The invasive ability of OS cells was evaluated by Transwell assay following peiminine treatment for 48 h. Scale bar = 100 μm. **(F)** Quantitative analysis of OS cell invasion rates (*n* = 3). **(G,J)** The expression of epithelial to mesenchymal transition (EMT)- and matrix metalloprotease (MMP)-associated markers was measured by western blot in OS cells following peiminine treatment for 48 h **(H,K)** Quantitative analysis of EMT-related proteins and MMPs (*n* = 3). *p < 0.05, ***p < 0.001.

We also examined the migratory and invasive abilities of OS cells after peiminine treatment. The results of the wound healing and Transwell assays showed that, compared with the control condition, the invasive and migratory capacity of OS cells was significantly reduced with peiminine treatment (Figures 7A–F). Because epithelial–mesenchymal transition (EMT) and matrix metalloproteases (MMPs) are reported to be associated with tumor invasion and metastasis, we subsequently measured the levels of EMT-related proteins and MMPs in control and peiminine-treated cells by western blotting.) The results showed that the expression levels of vimentin, MMP-2, and MMP-9 were decreased, and that of E-cadherin was increased with increasing peiminine concentrations (Figures 7G–K). In summary, these data showed that peiminine treatment could suppress the migration and invasion of OS cells.

### Peiminine Inhibited OS Growth *in Vivo*

**FIGURE 8:**
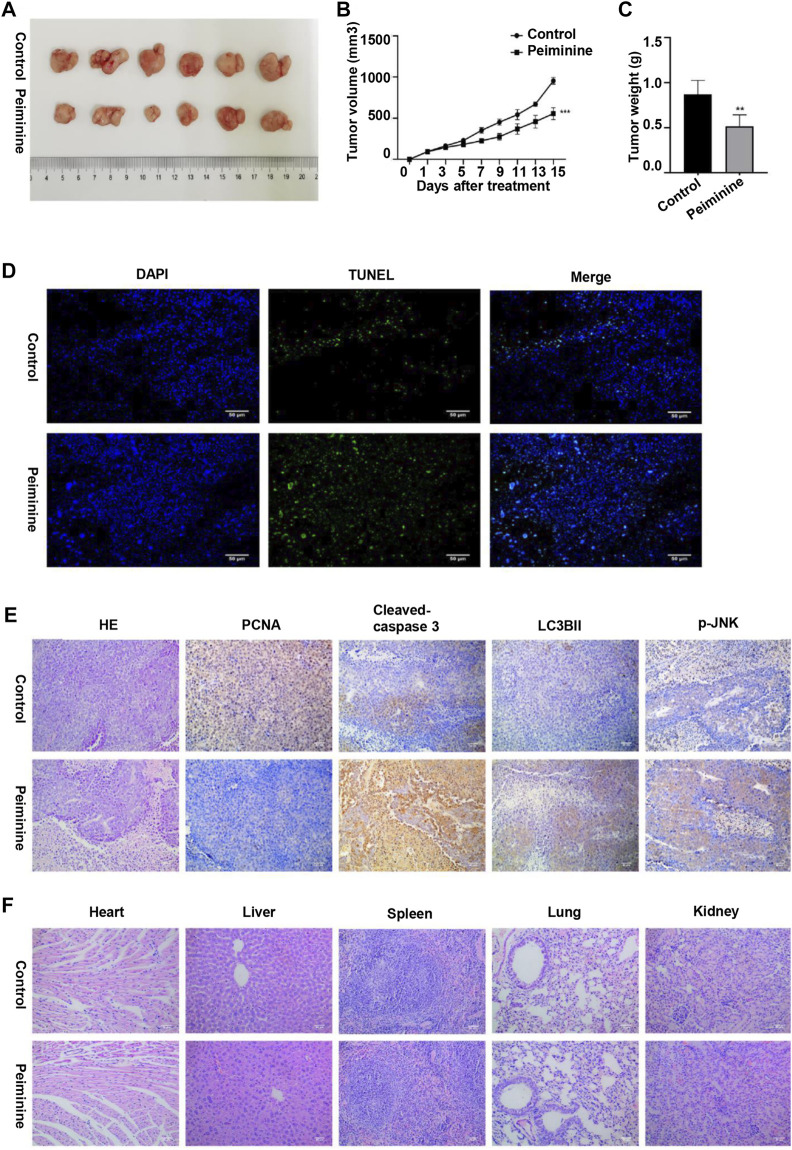
Peiminine inhibited osteosarcoma growth *in vivo*. **(A)** Representative images of the xenografts of MG-63 cells in control or peiminine-treated mice (*n* = 6). **(B)** Tumor volume was measured every other day after treatment. **(C)** Tumor weight was analyzed once all the tumors had been isolated. **(D)** Apoptosis in tumor tissues was determined by TUNEL assay. TUNEL‐positive cells are stained green and nuclei are stained blue (DAPI). **(E)** The expression of PCNA, cleaved caspase-3, LC3BII, and p-JNK was examined by immunohistochemistry. **(F)** Hematoxylin and eosin (H&E) staining was used to assess the histology of the major organs. Scale bars = 50 μm. **p < 0.01, ***p < 0.001.

To investigate the antitumor function of peiminine *in vivo*, we generated OS nude mouse xenograft model using MG-63 cells. Tumor volume and tumor weight analysis showed that peiminine significantly inhibited OS growth in the mice (Figures 8A–C). Moreover, no significant body weight loss was observed compared with that in mice in the control group (Supplementary Figure S2). Consistent with that seen *in vitro*, TUNEL staining results suggested that peiminine could induce the apoptosis of OS *in vivo* (Figure 8D). Immunohistochemical results further showed that cleaved caspase-3, LC3B-II, and p-JNK levels were significantly increased whereas that of proliferating cell nuclear antigen (PCNA) was decreased in the tumor tissues after peiminine treatment (Figure 8E). Moreover, histological examination of hematoxylin and eosin (H&E)-stained sections showed that peiminine did not cause major organ-related toxicity (Figure 8F). Overall, our findings clearly indicated that peiminine has strong antitumor activity with low toxicity *in vivo*.

## Discussion

OS is the most commonly diagnosed primary malignant bone tumor and is associated with a poor prognosis (Li et al., 2015b). Surgical resection combined with neoadjuvant chemotherapy is the standard treatment for OS (Durfee et al., 2016). However, resistance to current chemotherapeutic agents is common. Additionally, the chemotherapeutic regimens are associated with severe cardiotoxicity, which severely affects the prognosis of patients (Wang et al., 2016c). These observations highlight the acute need to identify novel drugs that target the malignant behavior of OS cells with high efficiency and fewer side effects. Over recent years, owing to their safety, long-term availability, and targeting potential, research attention has increasingly focused on herbal medicines, including peiminine (Zhao et al., 2018), as treatment options for a variety of cancers. However, the effects of peiminine on OS remain to be clarified, as do the putative underlying mechanisms. In this study, we demonstrated that peiminine inhibited proliferation, induced G0/G1-phase arrest, and led to apoptosis and autophagy through the ROS/JNK pathway in OS cells.

We found that peiminine significantly inhibited OS cell proliferation as determined by a CCK-8 assay. Moreover, a live/dead assay confirmed the antiproliferation activity of peiminine and further highlighted the morphological changes occurring in OS cells after peiminine treatment (Figure 1). Targeting the cell cycle, especially arrest at the G0/G1 phase, might be an efficient approach for inhibiting the excessive proliferation of cancer cells (Hanahan and Weinberg, 2011; Chen et al., 2013; Zhao et al., 2014). Cyclin D regulates the transition from the G1 to the S phase of the cell cycle by binding to cyclin-dependent kinase (CDK) (Vermeulen et al., 2003). Meanwhile, p27, a CDK suppressor protein, restricts the G1/S-phase transition by binding to cyclin D-CDK4 and cyclin E-CDK2 complexes (Slingerland and Pagano, 2000). Here, we found that peiminine induced G0/G1-phase arrest of OS cells *via* the regulation of cell cycle-related proteins (Figure 2).

Numerous drugs exert their antitumor functions by inducing cell apoptosis (Sun et al., 2004; Hu and Xiao, 2015). Peiminine induces apoptosis through both extrinsic and intrinsic apoptotic pathways in liver cancer, represses proliferation and growth of colorectal carcinoma by inducing autophagic cell death, and serves as an adriamycin chemosensitizer *via* the EGFR/FAK pathway in gastric cancer (Lyu et al., 2015; Tang et al., 2018; Chao et al., 2019). Similarly, in this study, the rate of OS cell apoptosis was significantly increased after peiminine treatment. We also investigated the putative mechanisms involved in how peiminine induces the apoptosis of OS cells. It is well known that apoptosis includes both the death ligand and the mitochondrial pathways (Huang and Oliff, 2001). The mitochondrial pathway relies on the loss of mitochondrial membrane potential and an increase in membrane permeability (Siegel and Lenardo, 2002). Upon the induction of cell apoptosis, Bax oligomerizes and translocates from the cytoplasm to the mitochondrial membrane, where it enhances membrane permeability by interacting with pore proteins in the mitochondrial membrane (Marzo et al., 1998; Narita et al., 1998). Bcl-2 plays a prosurvival role by inhibiting the release of mitochondrial cytochrome C under various proapoptotic stimuli (Murphy et al., 2000). In this study, following peiminine treatment, OS cells experienced mitochondrial membrane depolarization, an increase in the Bax/Bcl-2 ratio, finally resulting in the activation of caspase-3 and −9 (Figure 3). TUNEL assay and immunohistochemistry of mouse tumor tissues revealed that, compared with controls, the rate of apoptosis and the levels of proapoptotic proteins were significantly increased following peiminine treatment (Figure 8). These results suggested that peiminine induces apoptosis in OS cells *via* the mitochondrial pathway.

Given the reported role of autophagy in tumor cell death, we explored whether peiminine induced autophagy in OS cells and whether it mediated the peiminine-induced death of OS cells, at least in part (Zhu et al., 2017). In this study, we analyzed autophagy by observing autophagosomes by TEM, which is the gold standard for detecting autophagy; monitored the autophagic flux, which assesses the whole process of autophagy; and measured the expression of p62, a marker of autophagic degradation. Compared with that previously reported (Zhao et al., 2018), we found that the expression of p62 was downregulated following peiminine treatment. Our results further revealed that peiminine enhanced autophagosome formation and promoted autophagic flux in OS cells. Numerous studies have stressed the double effect of autophagy in cancer (Sui et al., 2013; Duffy et al., 2015). To verify the effect of peiminine-induced autophagy on OS, we performed CCK-8 assays and western blot in the presence of CQ. The results revealed that CQ rescued the inhibition of cell viability and the increase in the levels of proapoptotic proteins induced by peiminine, suggesting that peiminine-induced autophagy is involved in OS cell death (Figure 4).

Some studies have demonstrated that ROS is a key regulator of cell fate (Zou et al., 2017). Basal ROS levels contribute to cell proliferation and viability; however, excessive ROS production can result in damage to cellular components, leading to apoptosis and autophagy (Maiuri et al., 2007). In this study, consistent with previous reports, the peiminine-induced effects on intracellular ROS production could be better observed using live-cell imaging. The cell contours and subcellular structures were more clearly demarcated and ROS production sites could be identified, allowing for a more comprehensive evaluation. The results revealed that peiminine treatment markedly increased ROS levels in OS cells, while NAC pretreatment could block peiminine-induced apoptosis and autophagy, suggesting that these effects of peiminine were dependent on increased intracellular ROS generation. ROS can function as a second messenger, activating a variety of downstream molecules, mainly members of the MAPK family, including JNK, p38, and ERK1/2 (Zhang et al., 2013; Zhang et al., 2015; Mi et al., 2016). Costunolide can activate the ROS/MAPK pathway in renal cell carcinoma (Fu et al., 2020), honokiol can activate the ROS/ERK1/2 pathway in OS cells (Huang et al., 2018), and metformin can activate the ROS/JNK pathway in OS cells (Li et al., 2020). In line with previously reported results, in the present study, we found that peiminine treatment activated JNK, whereas NAC treatment abrogated JNK phosphorylation. Furthermore, both SP600125 and NAC could suppress peiminine-induced apoptosis and autophagy. These results demonstrated that peiminine induces apoptosis and autophagy in OS cells through the ROS/JNK pathway (Figures 5, 6).

Early invasion and migration of OS cells severely affect the prognosis of patients (Jin et al., 2020). Therefore, we investigated for the first time the impact of peiminine on the invasive and migratory ability of OS cells. The results revealed that peiminine markedly reduced the invasive and migratory potential of OS cells and modulated the levels of invasion- and migration-related proteins (Figure 7).

**FIGURE 9:**
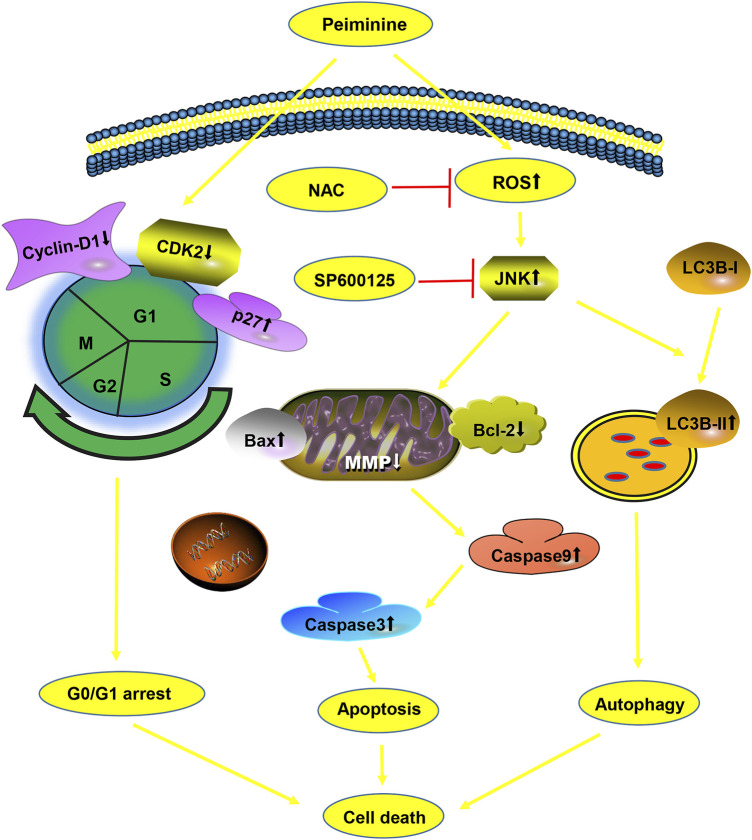
A diagram of the effects of peiminine on osteosarcoma (OS) cells.

In conclusion, we demonstrated for the first time the antiosteosarcoma activities of peiminine and elucidated the potential underlying mechanism. We found that peiminine treatment could promote G0/G1-phase arrest, induce apoptosis and autophagy through the ROS/JNK pathway, and inhibit the invasion and migration of OS cells (Figure 9). Our study established an experimental basis for the use of peiminine as a potential drug candidate for the treatment of OS.

## Data Availability

The raw data supporting the conclusions of this article will be made available by the authors, without undue reservation.
