# Supplementary material for: Peiminine Induces G0/G1-Phase Arrest, Apoptosis, and Autophagy via the ROS/JNK Signaling Pathway in Human Osteosarcoma Cells in Vitro and in Vivo
Source: Front Pharmacol. 2021 Nov 12;12:770846. doi: 10.3389/fphar.2021.770846 (PMC8633898; doi:10.3389/fphar.2021.770846)
Supplement: Supplementary file 1 [file Table1.docx]

**The IC50 values of peiminine on osteosarcoma cells**

|  | **MG-63** | **Saos-2** |
| --- | --- | --- |
| **24h (μM)** | **412** | **483** |
| **48h (μM)** | **178** | **195** |
| **72h (μM)** | **113** | **121** |
