# Supplementary figures and images for: Peiminine Induces G0/G1-Phase Arrest, Apoptosis, and Autophagy via the ROS/JNK Signaling Pathway in Human Osteosarcoma Cells in Vitro and in Vivo
Source: Front Pharmacol. 2021 Nov 12;12:770846. doi: 10.3389/fphar.2021.770846 (PMC8633898; doi:10.3389/fphar.2021.770846)

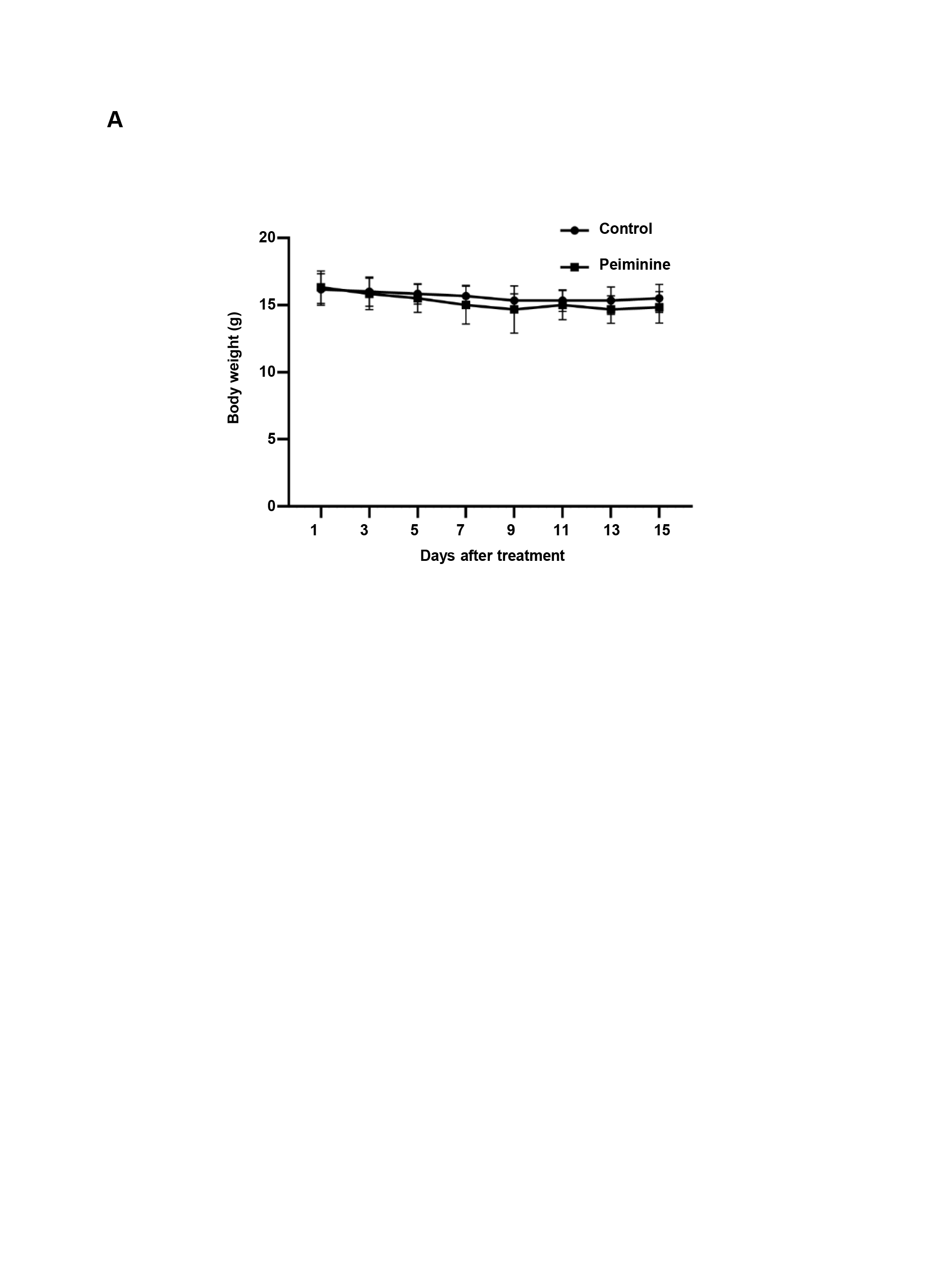

Supplement: Supplementary file 2 [file Image2.TIF]

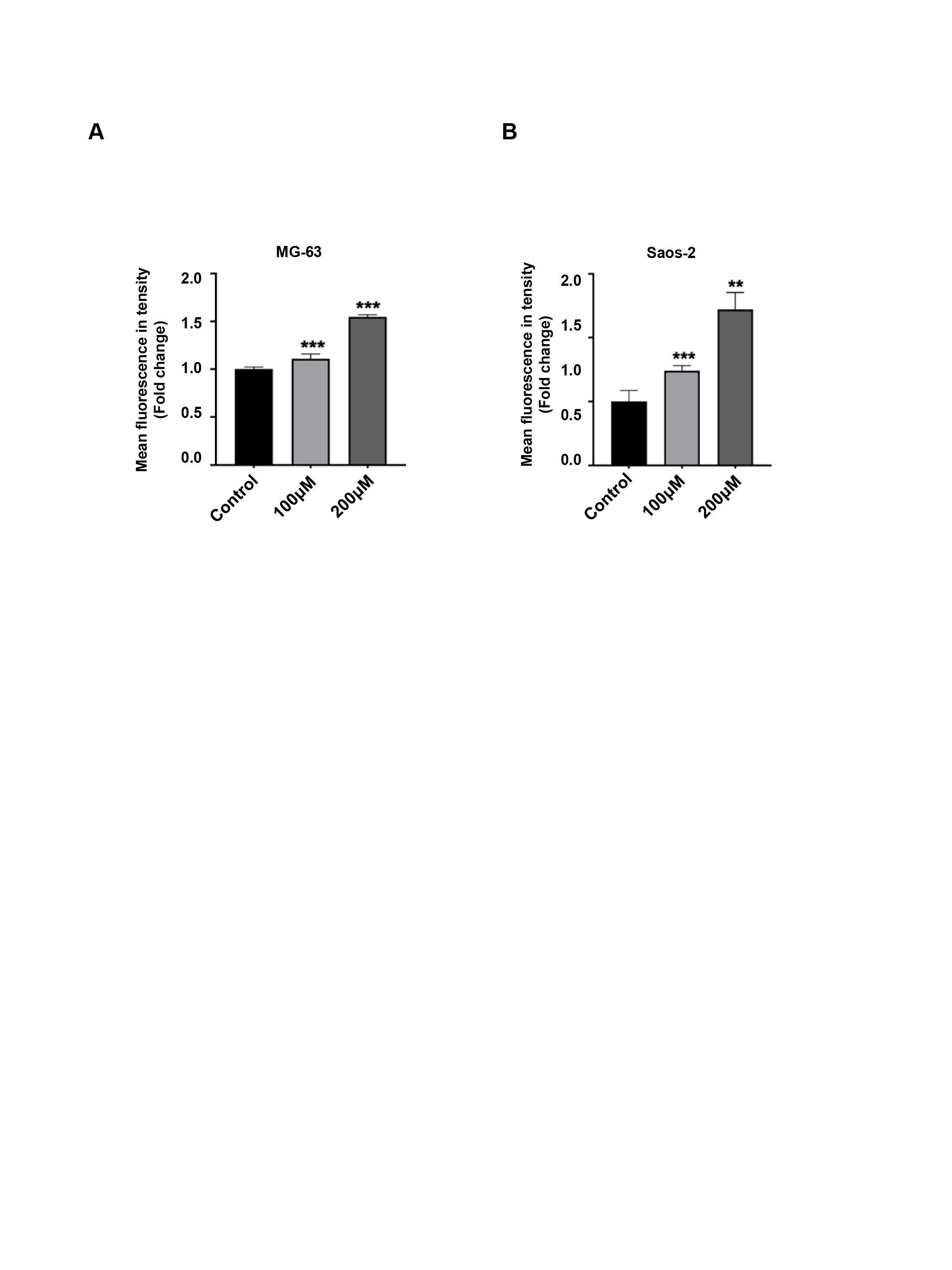

Supplement: Supplementary file 3 [file Image1.TIF]
