# Supplementary material for: Peiminine Induces G0/G1-Phase Arrest, Apoptosis, and Autophagy via the ROS/JNK Signaling Pathway in Human Osteosarcoma Cells in Vitro and in Vivo
Source: Front Pharmacol. 2021 Nov 12;12:770846. doi: 10.3389/fphar.2021.770846 (PMC8633898; doi:10.3389/fphar.2021.770846)
Supplement: Supplementary file 4 [file DataSheet1.docx]

**Supplementary Figure 1 |** Peiminine induces ROS generation.

**(A, B)** The quantitative analysis Intracellular ROS levels were measured by fluorescence microscope with DCFH-DA staining. (***P* < 0.01, ****P* < 0.001).

**Supplementary Figure 2 |** Peiminine inhibits growth of osteosarcoma in vivo.

**(A)** Body weights were measured every other day.
